# A Chatbot (Juno) Prototype to Deploy a Behavioral Activation Intervention to Pregnant Women: Qualitative Evaluation Using a Multiple Case Study

**DOI:** 10.2196/58653

**Published:** 2024-08-14

**Authors:** Elisa Mancinelli, Simone Magnolini, Silvia Gabrielli, Silvia Salcuni

**Affiliations:** 1 Department of Developmental and Socialization Psychology, University of Padova Padova Italy; 2 Digital Health Lab, Centre for Digital Health and Wellbeing, Fondazione Bruno Kessler Povo, Trento Italy; 3 Intelligent Digital Agents, Centre for Digital Health and Wellbeing, Fondazione Bruno Kessler Povo, Trento Italy

**Keywords:** chatbot prototype, co-design, pregnancy, prevention, behavioral activation, multiple case study

## Abstract

**Background:**

Despite the increasing focus on perinatal care, preventive digital interventions are still scarce. Furthermore, the literature suggests that the design and development of these interventions are mainly conducted through a top-down approach that limitedly accounts for direct end user perspectives.

**Objective:**

Building from a previous co-design study, this study aimed to qualitatively evaluate pregnant women’s experiences with a chatbot (Juno) prototype designed to deploy a preventive behavioral activation intervention.

**Methods:**

Using a multiple–case study design, the research aims to uncover similarities and differences in participants’ perceptions of the chatbot while also exploring women’s desires for improvement and technological advancements in chatbot-based interventions in perinatal mental health. Five pregnant women interacted weekly with the chatbot, operationalized in Telegram, following a 6-week intervention. Self-report questionnaires were administered at baseline and postintervention time points. About 10-14 days after concluding interactions with Juno, women participated in a semistructured interview focused on (1) their personal experience with Juno, (2) user experience and user engagement, and (3) their opinions on future technological advancements. Interview transcripts, comprising 15 questions, were qualitatively evaluated and compared. Finally, a text-mining analysis of transcripts was performed.

**Results:**

Similarities and differences have emerged regarding women’s experiences with Juno, appreciating its esthetic but highlighting technical issues and desiring clearer guidance. They found the content useful and pertinent to pregnancy but differed on when they deemed it most helpful. Women expressed interest in receiving increasingly personalized responses and in future integration with existing health care systems for better support. Accordingly, they generally viewed Juno as an effective momentary support but emphasized the need for human interaction in mental health care, particularly if increasingly personalized. Further concerns included overreliance on chatbots when seeking psychological support and the importance of clearly educating users on the chatbot’s limitations.

**Conclusions:**

Overall, the results highlighted both the positive aspects and the shortcomings of the chatbot-based intervention, providing insight into its refinement and future developments. However, women stressed the need to balance technological support with human interactions, particularly when the intervention involves beyond preventive mental health context, to favor a greater and more reliable monitoring.

## Introduction

### User-Centered Design of Digital Mental Health Interventions

eHealth is a burgeoning field that integrates medical informatics, public health, and business. It encompasses delivering health services and information through the internet and digital technologies. In this domain, e-mental health specifically focuses on leveraging technologies, such as smartphone apps, websites, chatbots, and virtual reality, to enhance and support mental health care [1-3]. e-Mental health holds many advantages, including the increased scalability of mental services, in terms of screening, prevention, and treatment, leading to reduced costs for the broader health care system [4-6]. However, while the potential benefits of digital technology can be considerable, their actual implementation and use, especially within the field of e-mental health, often fall short. The journey from preuse considerations to initial adoption and, crucially, sustained use poses challenges that need careful navigation and understanding. In this regard, a recent review [7] exploring design methods and approaches for digital tools in mental health emphasized that human-centered design methods, thus those focusing on user experience (UX) rather than just engineering design, are not fully integrated into the field. The reported design approaches are predominantly external, lacking the perspective of the end users for whom the tool is intended. Indeed, when developing digital solutions, it is essential to consider 4 key components: the design issue and solution, the context in which the design occurs, the dynamics and organization of the design activity, and the actors contributing to the design [8-10]. Within the context of e-mental health intervention, the above altogether emphasizes the significance of co-design, a collaborative process strongly involving targeted end users to contribute to all stages of e-mental health intervention development. This inclusive approach encompasses needs assessment, content development, pilot-testing, and finally, dissemination [11]. The Obesity-Related Behavioral Intervention Trials (ORBIT) model [12] is instrumental to this end. The ORBIT model, which uses a user-centered design, provides a methodological framework encompassing a pliable and iterative progressive procedure, predefined clinically significant milestones for advancement, and the option to revert to a prior phase of refinement in case of suboptimal outcomes. Its primary emphasis is on pre-efficacy development and testing, yet not failing to incorporate subsequent research phases to illustrate that treatment optimization is viable even for interventions that have attained the efficacy or effectiveness stage [12].

### e-Mental Health in Perinatal Care: A Focus on Prevention Interventions

The World Health Organization (WHO) [13] has consistently emphasized the significance of identifying and preventing risks, with the WHO and the United Nations Population Fund acknowledging maternal mental health as a pivotal factor in accomplishing the Millennium Development Goals [14]. The transition to motherhood involves various intrapersonal and interpersonal changes and challenges that can have negative effects on women’s mental health, increasing the risk of developing peripartum depression [15-17]. However, despite the negative repercussions this poses on the women, the child, and the whole family [18], as well as the broader society [19-21], it often goes untreated. There are various reasons for this. On the one hand, few women proactively seek professional assistance for their mental health problems, mainly due to factors such as lack of mental health literacy; stigma; and practical barriers like childcare, professional, and financial constraints [22]. By contrast, women face limited access to specialized perinatal mental health services, which is attributed to the capacity constraints of existing services and long waiting times for those in need of support [23,24]. Therefore, many women never receive any support or treatment. Indeed, this situation has sparked interest in the potential of e-mental health. It can circumvent some of the aforementioned barriers, ultimately facilitating a more widespread help-seeking process; this has led to the creation and dissemination of scalable and more far-reaching tools to support the well-being and mental health of perinatal women [25,26]. In this context, the stepped-care model is noteworthy, as its intentions are focused on promoting the dissemination of mental health programs by facilitating coordination between primary and secondary mental health services [27], and this coordination can be facilitated through e-mental health. This would ultimately align with the evidence that engaging in help-seeking behaviors increases the likelihood of perinatal women seeking further assistance for their depression symptoms [28]. In this regard, structured, evidence-based interventions such as behavioral activation (BA) might be particularly suitable. BA is a behavioral intervention designed to alleviate symptoms of depression [29-32] by offering individuals practical strategies to improve their adjustment and well-being and supporting participation in enjoyable and positive activities while reducing engagement in behaviors that worsen depressive symptoms [29,33]. As such, these interventions hold great potential as initial broad-case preventive work. However, when specifically focusing on peripartum depression, there appears to be a deficiency in digital prevention and treatment programs at large [34], and of BA interventions as well [35], in addressing depression symptoms during pregnancy compared with the postpartum period, thus underscoring the necessity to boost the development and evaluation of primary mental health services.

### This Study: Within the Iterative Design Phase

This study arises from the results obtained by a previous exploratory co-design study [36] investigating the feasibility of an internet-based BA intervention for pregnant women showing subclinical symptoms of depression. As such, it constitutes the second phase of investigation within the “design phase” foreseen by the above-reported ORBIT model [12]. This prior exploratory study not only aimed to assess the initial feasibility of the intervention but also sought to gather valuable feedback directly from pregnant women. This then guided the adjustment of the intervention’s content and structure while promoting the use of a different digital solution. More specifically, the study aimed to compare a guided and unguided version of the digital intervention, with the guided group involving psychologists who engaged in weekly text message conversations with women to support them in the intervention content revision. In this respect, data suggested that the guided group showed greater adherence and were more willing overall to finish the intervention than the unguided group. Building on this and in line with the existing literature [37,38] highlighting the potential benefit of including chatbots within psychological interventions by fostering intervention adherence through increased engagement and involvement, a new structuring of the BA intervention as a chatbot-delivered intervention was prototyped. Chatbots are artificial intelligence–enabled engagement technologies, falling under the category of technologies that enable interaction with patients through natural language processing by engaging in limited text conversations intending to support subsequent behavior-change tasks [39]. It is crucial to emphasize that in this context, chatbots are conceptualized as tools suitable for educational purposes, facilitating the acquisition of specific evidence-based techniques or skills [40] resulting in suitability for application in preventive contexts.

Mindful of the above, this study aims to qualitatively evaluate, through a multiple case study, pregnant women’s experience and perception of a chatbot prototype to deploy a BA preventive support tool and intervention. In this regard, incorporating a dedicated prototype evaluation during co-design can streamline the process of conducting rigorous evaluations in real-world settings during the subsequent evaluative phases, which may involve activities such as pilot-testing and subsequent randomized controlled trials [41]. Furthermore, women’s desire for improvement and technological advancements of chatbot-based technology in the field of perinatal mental health was also investigated. As such, this study bounds the design and evaluation of the chatbot and prevention intervention it deploys within the ORBIT methodological framework [12], in favor of a thorough and meticulous evaluation of the intervention design phase regarding both definition and refinement. In line with this, a multiple–case study design is used as it permits the conduct of a comparative analysis of cases, aiming to identify both similarities and differences among them and, thus, in the perception of the chatbot and the content it deploys. In addition, this approach seeks to unveil patterns and themes that arise from the cross-case analysis. By evaluating the phenomenon of interest across different contexts, a multicase study might enhance the validity of findings by investigating in depth how the phenomenon may vary or remain consistent under various circumstances [42].

## Methods

### Ethical Considerations

Ethical considerations adhered to the guidelines outlined in the Declaration of Helsinki [43] and European data protection laws (EU GDPR 679/2016). Approval for the study was obtained from the Ethical Committee of the Psychology Department at the University of Padova (approval 5434/2023). Participants provided their informed consent to participation and data publication for scientific reasons.

### Participants and Enrolment Procedure

Women aged >18 years and between the 12th and 30th week of gestation could take part in this study. Exclusion criteria were the following: clinically significant depression symptoms (Patient Health Questionnaire-9 [PHQ-9] [44] score≥15), suicidal ideation (PHQ-9 item 9), present or past history of psychiatric disorders, and experiencing an artificially induced pregnancy. To allow participation, a Google Form link containing the baseline questionnaires was shared through social media platforms (ie, Facebook and Instagram) in pregnancy-related national groups and pages. After the inclusion and exclusion criteria evaluation, women were provided with the information needed to start the interaction with the chatbot in Telegram and sent a copy of the informed consent they had agreed on that was reported within the web-based questionnaire. To uphold confidentiality, each participant was assigned a unique alphanumeric code. Women were granted the autonomy to withdraw from participation at any point without the obligation to provide reasons and without facing any adverse consequence. Furthermore, they were clearly informed that the software (ie, Telegram and the chatbot) did not constitute a medical device, as its use does not extend to the diagnosis, prevention, monitoring, prediction, prognosis, treatment, or alleviation of diseases. It was, instead, clarified that the developed support intervention and related software were exclusively intended for research purposes and used for the sole collection, storage, transmission of data and administration of questionnaires.

A total of 12 women completed the baseline questionnaire. Among them, 2 dropped out after the first interaction (week 1), 2 after completing the interaction in week 2, and 2 following the third interaction (week 3). One participant withdrew after completing the interaction in week 4. Among those who dropped out in the early weeks, 5 reported medical conditions: Crohn disease, risk of miscarriage associated with a shortened cervix and hypertonic pelvic floor, gestational diabetes, hypothyroidism, and fibroma. Ultimately, 5 participants were included in the multiple–case study evaluation, with none reporting any medical conditions. Of them, 4 (80%) participants reached the postintervention questionnaire evaluation, while 1 (20%) had to interrupt the interaction after week 4; however, she agreed to participate in the final semistructured interview. Given that this study aimed to qualitatively evaluate the perception and experience with a chatbot prototype, the decision was made to include this participant despite not finishing the study since she nonetheless was able to engage with the chatbot for more than half of the anticipated interactions.

### The Intervention Content and Structuring

This study aligns with the iterative process outlined in the ORBIT model [12] for intervention design and evaluation. Specifically, it falls within the refined subphase of the initial design phase, in which practical aspects such as mode and agent of delivery, as well as the frequency and duration of contact, are evaluated to identify the most efficient ways to achieve clinical targets. Parallel to this, and in reference to the Digital Product Lifecycle, we care to emphasize that this project is at the beginning stages of the product life cycle, thus moving back and forth between the “definition phase” (in which the product or intervention concepts and related digital requirements are defined) and the “design phase” (which involves prototyping and pilot-testing the product) [45,46].

**Figure 1:**
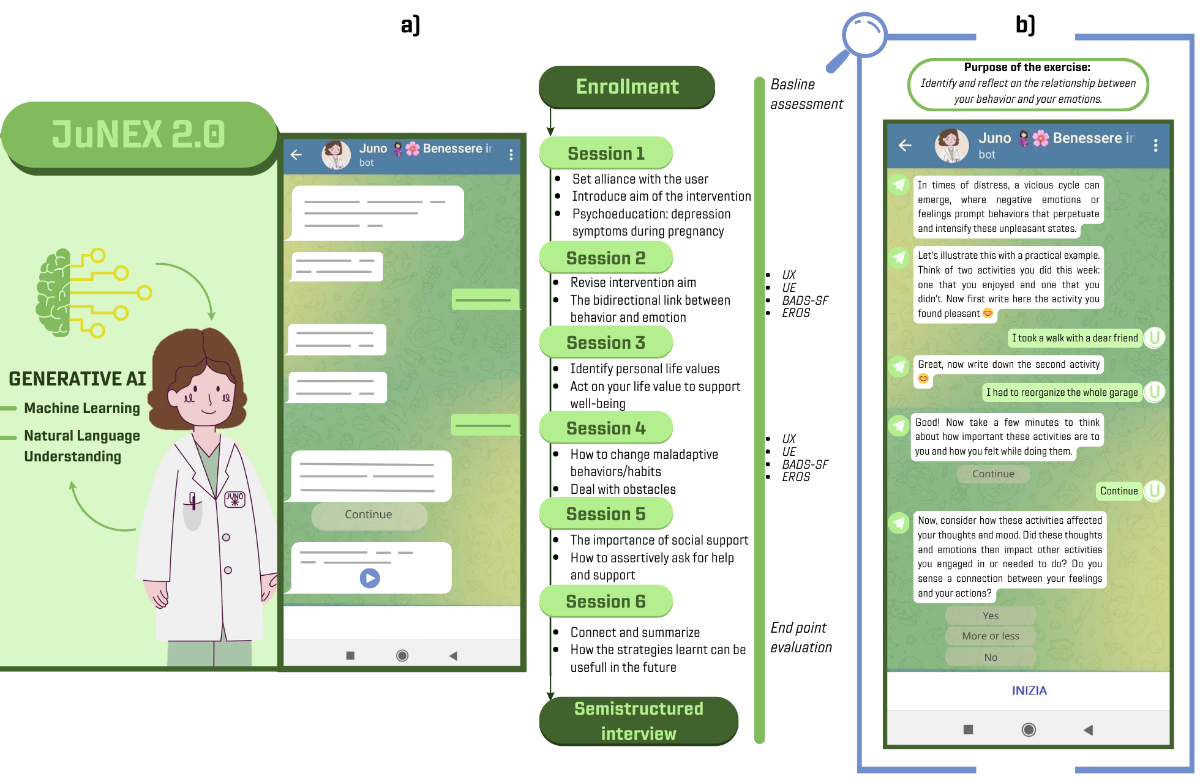
Chatbot Juno on Telegram. (a) Intervention structure and (b) an example of interaction between Juno and the user. The images recreate the interface seen by participants and show the appearance of Juno within the illustrative videos and images. In B, the reported example of an interaction depicts a simulation of an exercise part of the topics of week 2, namely "The bidirectional link between behavior and emotions". AI: artificial intelligence; BADS-SD: Behavioral Activation for Depression Scale-Short Form; EROS: Environmental Reward Observation Scale; UE: user engagement; UX: user experience.

Accordingly, this study focuses on evaluating a revised version of an intervention based on an evidence-based BA intervention protocol (behavioral activation treatment for depression-revised) [47]. This revised intervention represents a second evaluation that builds upon exploratory testing conducted in a preceding co-design study [36]; as such, thorough information on the intervention content and rationality can be found in this prior study paper. However, commencing with the results from this latter study, in this study, the intervention was organized into 6 weekly sessions (Figure 1A), omitting the 3 additional ones previously included. The intervention content was streamlined by eliminating separate in-between–session homework. Instead, the essential components of the homework were incorporated within the main sessions or interactions as on-the-moment exercises strategically designed to promote the original intent of the homework. In this context, it is noteworthy that while the original protocol may not explicitly encompass a comprehensive functional analysis, several treatment components seamlessly aligned within such a framework and were further enhanced in the modified version of the intervention. This alignment is underscored by BA’s dual objectives of pinpointing factors that sustain or reinforce depressive behaviors (both positive and negative reinforcement) and identifying positive reinforcers that can support healthy behavioral patterns (Figure 1A). This process thus forms the basis for understanding the functional aspects of behavior, laying the groundwork for targeted strategies that can aid the person in autonomously addressing and modifying the maladaptive behavioral patterns effectively.

Moreover, there was a modification in the mode of delivering the intervention. Specifically, the content, previously structured as an e-learning course, was facilitated through a rule-based chatbot named Juno, operationalized within the Telegram platform with the sole purpose of delivering the intervention. As such, information was delivered through text messages, complemented by explanatory videos and images using the Telegram interface. The text messages were sent by Juno, which adhered to a preestablished protocol that had to be followed sequentially, enabling structured dialogues in which women engaged primarily by selecting the predefined buttons to navigate the conversation. Due to the rule-based nature of Juno, individualized feedback was not provided. In this regard, Figure 1B depicts a simulation of the interaction between Juno and the user, showing how Juno responds and guides the user during the on-the-moment exercises (the reported example is an exercise conducted during week 2 reported in Figure 1A “The bidirectional link between behavior and emotions”).

Moreover, by using the Telegram interface, participants can access multimedia resources such as videos and images in the multimedia section of the app. In addition, they could scroll back through the chat history to review past topics, although there was not a specific page summarizing the weekly intervention topics. This allowed participants to revisit and reinforce previous discussions as needed while maintaining the logical sequencing of the intervention. Conversations were structured to last around 10 minutes per session.

### The Implementation of the Chatbot Juno in Telegram

The chatbot Juno was developed drawing inspiration from the methodology used in designing Motibot, a chatbot dedicated to providing psychosocial support to adults with diabetes mellitus [48]. Leveraging the capabilities of the Rasa open-source platform [49], which has been explicitly tailored for chatbot development and training, became viable owing to the domain-agnostic nature of Motibot’s core structure, which provided a remarkably flexible foundation. The Rasa platform seamlessly integrates advanced machine learning techniques and harnesses pretrained embeddings from language models. This integration empowers the construction of a chatbot finely tuned to a specific language. The synergy of machine learning techniques with crafted rules ensures a chatbot that is not only dynamic but also highly responsive. Within Juno, the pivotal role played by natural language understanding [50] became evident in interpreting user messages while considering the conversational history. A carefully defined set of variables facilitated a smooth transition between turns in the dialogue.

For instance, named entity recognition, a specific natural language understanding task, was used to interpret the intent “say your name” and identify the entity “user’s name.” Juno optimally used Telegram as its user interface, offering numerous advantages to users while streamlining the development process. In addition, Telegram’s built-in support for interactive tools, including buttons, links, and images, enhanced the overall UX. In this regard, enhancing UX involves using personalized interaction time frames. Juno, as part of its intervention process, prompted users at the end of the initial day to specify when they prefer follow-up contacts. This proactive approach assisted users in scheduling their intervention; Juno uses Rasa’s reminder interface to accomplish this task. However, potential server malfunctions can affect this tool. To mitigate such issues, Juno allows users to initiate the interaction (eg, by writing the message “Can we start?”) if the reminder date passes without any notification. Despite being a solution to a possible interaction problem, this approach should maintain a positive UX. Furthermore, it should be acknowledged that in this initial phase of development, the possibility that the chatbot could occasionally overlook an appointment was a possibility.

Moreover, in line with what was reported above, it is noteworthy that Juno follows an expert-written structured script to maintain focus on the intervention content and avoid deviating from the intended topics. Users can provide input by selecting predefined buttons or providing written responses, but they do not receive personalized feedback based on their input. If users attempt to engage with Juno outside the scope of the intervention, Juno informs them that it cannot respond to such queries and returns to the predefined interaction by starting from where they had left off.

With regard to data storage, no further development was required, as the native support of Rasa for storing interactions in a MongoDB database (ie, a universal time stamp) ensures both consistency and the archiving of users’ data (ie, log-in information, the time spent by each user interacting with Juno, etc).

### Measurement Instruments

#### Overview

During the baseline assessment, women were asked the following demographic information: age, gestational week, if the pregnancy was physiological or induced through medically assisted techniques, marital status, educational level, category of occupation, living location, past and present psychiatric history, and presence of any medical condition (both pregnancy-related and not). Moreover, during the baseline assessment, participants completed questionnaires assessing psychological symptoms, levels of BA, and perceived environmental reward. The same questionnaires were administered at the end of the sixth week of interactions, facilitated by Juno in the Telegram Chat, for a postintervention evaluation; the UX and user engagement (UE) measures were included in the postintervention assessment.

#### Psychological Symptoms

Depression symptoms were evaluated through 2 unidimensional self-report tools: the PHQ-9 [44,51] and the Edinburgh Postnatal Depression Scale [52,53]. The PHQ-9 assesses the severity of depression symptoms over the past 2 weeks through 9 items measured on a 4-point Likert scale (0=“not at all”; 3=“almost every day”). Items align with the diagnostic criteria of the *Diagnostic and Statistical Manual of Mental Disorders*, *Fourth Edition* [54]. A score of ≤9 indicates mild or no depression symptoms, between 10 and 14 indicates moderate symptoms, and ≥15 indicates severe symptoms. Item 9 specifically assesses suicidal ideation. The Edinburgh Postnatal Depression Scale also assesses the severity of depression symptoms, yet on the previous week and more specifically in association with the perinatal period. It comprises 10 items measured on a 4-point Likert scale (0=“no, not at all”; 3=“yes, always”), with item 10 assessing suicidal ideation. Scores range between 0 to 30, and a score of ≥13 suggests probable depression. Anxiety symptoms were measured through the Generalized Anxiety Disorder-7 [51,55], a unidimensional self-report tool gauging the severity of these symptoms over the past 2 weeks through 7 items measured on a 4-point Likert scale (0=“never”; 3=“almost every day”). Scores range between 0 and 21; a score between 0 and 4 suggests minimal anxiety, between 5 and 9 mild anxiety symptoms, between 10 and 14 moderate anxiety symptoms, and ≥15 severe anxiety symptoms. Finally, stress symptoms were assessed through the Perceived Stress Scale-10 [56,57], a unidimensional self-report tool assessing stress symptoms over the past month using 10 items measured on a 4-point Likert scale (0=“never”; 3=“quite often”). Scores range between 10 and 40, with scores ranging from 0 to 13 suggesting lower stress levels, between 14 and 26 moderate stress levels, and ≥27 high perceived stress levels.

#### BA Measures

The BA for Depression Scale-Short Form [58] was used to measure changes in avoidance and activation during BA interventions for depression over the past week. It is a self-report featuring 9 items measured on a 7-point Likert scale (0=“not at all”; 6=“completely”), providing scores for BA, behavioral avoidance, and a total score ranging from 0 to 54. The Environmental Reward Observation Scale [59], a unidimensional self-report tool, was also used; it measures the level of environmental reward perceived in recent months through 10 items rated on a 4-point Likert scale (1=“strongly disagree”; 4=“strongly agree”). Scores range from 10 to 40.

#### UX and UE

The UX was evaluated through the Mobile Application Rating Scale [60], a self-report tool evaluating the quality of an app and its features. Comprising 23 items scored on a 5-point Likert scale (1=“poor”; 5=“excellent”), it assesses 4 dimensions of objective quality: engagement, functionality, esthetics, and information, along with a subjective quality scale. Only subscales related to “information,” “subjective app quality,” and “app-specific” (function) were considered for this study, totaling 17 items. UE was instead evaluated through the User Engagement Scale-Short Form [61], a short self-report tool assessing UE with a digital solution. With 12 items based on a 5-point Likert scale (1=“strongly disagree”; 5=“strongly agree”), it encompasses factors such as focused attention, perceived usability, esthetic attractiveness, and reward. Higher scores index a more positive evaluation.

#### Semistructured Interviews

Semistructured interviews, conducted by the first author between October and December 2023, featured 15 main questions tailored to the study. The semistructured interview comprehends 3 main blocks of questions and related probing questions: one focused on women’s personal experience with the intervention content (4 questions), another focused on their experience with the chatbot and the overall platforms (5 questions), and the last one inquired on opinions for future technological advancements (6 questions). Before asking the last block of questions, participants were provided with the definitions of *digital intervention* and *technological advancement* within the context of chatbot technologies, as reported in the Multimedia Appendix 1. The interviews were conducted approximately 10 to 14 days after the participants had finished the interactions with the chatbot Juno; they were conducted either by phone call or through Google Meet, based on the participants’ preference. With the participants’ consent, the interviews were audio recorded for transcription and evaluation.

### Data Analysis

All the analyses were computed with RStudio (RStudio IDE). Participants’ questionnaire scores were assessed, and score differences in psychological symptoms and activation levels, between preintervention and postintervention time points, were calculated by subtracting the postintervention scores from the baseline ones. Relying on the qualitative meaning of response points (particularly for the psychological symptoms, measured on a 4-point Likert scale), differences between time points were commented on when they differed by a minimum of +3 or –3 score points.

The semistructured interviews were evaluated in 2 different but complementary manners. First, a purely qualitative descriptive evaluation was conducted by extracting and evaluating the key points reported by each case in the related interview transcript for each question. Subsequently, a text-mining analysis was performed using the R package *quanteda* [62]. To this end, (1) the transcripts, written in Italian, were tokenized by using the specific *quanteda* function and converting uppercase letters into lowercase letters, removing numbers, punctuation, and stop words. Subsequently, (2) user responses were subdivided and grouped based on the question they referred to in separate .txt files. Finally, (3) recurrent words (ie, word stems) and their diagrams (ie, pairs of reoccurring word stems) were extracted; the former were considered recurrent if they appeared at least 3 times, while the latter if they appeared at least 2 times across interviews. The 3-occurrences criterion threshold was defined in line with past research [48]. In particular, the 3-occurrences criterion for including a stem was chosen based on the assumption that through this, an occurrence is expected to belong to the 5% most recurrent ones. This criterion resulted in the extraction of between 2.4% and 9.2% of the most recurrent stems (average 5.5%) for the different questions, reasonably complying with the assumed 5% threshold. In addition, for a given question, the average occurrence of stems was 1.3; thus, a 3-occurrences threshold was equivalent to the condition of a stem recurring with a frequency corresponding to more than twice the average occurrence.

## Results

### Cases Presentation

**Figure 2:**
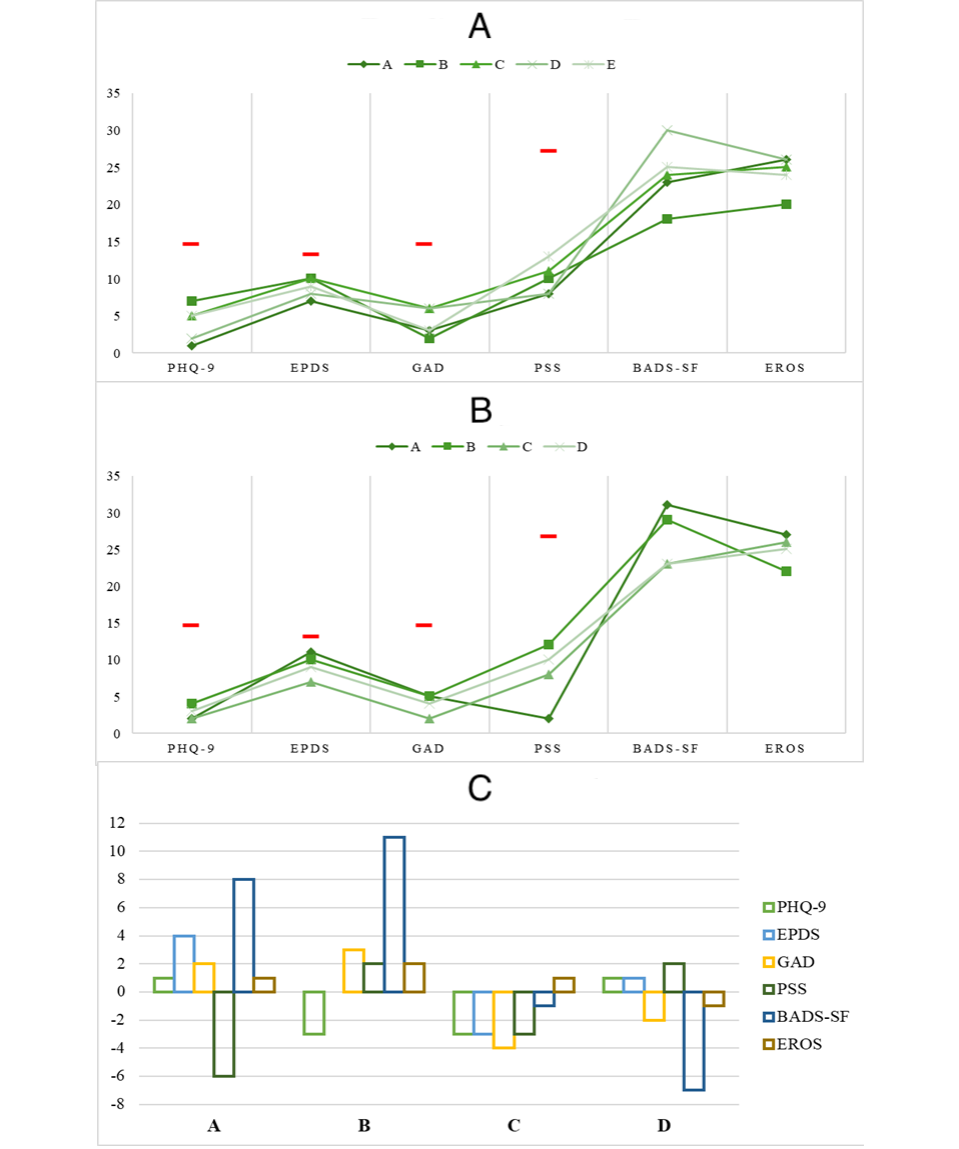
Participants’ symptoms and activity levels at baseline and postintervention time point and the difference between the 2 time points. In A and B, the red dashes index the clinical scales’ cutoff (see the Psychological Symptoms in the Measurement Instruments section). BADS-SF: Behavioral Activation for Depression Scale-Short Form; EPDS: Edinburgh Postnatal Depression Scale; EROS: Environmental Reward Observation Scale; GAD-7: Generalized Anxiety Disorder-7; PHQ-9: Patient Health Questionnaire-9; PSS: Perceived Stress Scale.

Table 1 shows the participant’s demographic information. Figure 2 shows their scores regarding psychological symptoms, activity level, and environmental reward at baseline (Figure 2A) and the postintervention time point (Figure 2B), further plotting the difference between the 2 time points (Figure 2C). Of the 5 cases, participant E completed the interaction with Juno until (and including) week 4 because of a technical issue with the server provider of the chatbot (the update of the server’s public certificate resulted in a compromised connection between the Rasa server and Telegram, and despite efforts within the support time frame, communication restoration was unsuccessful). As such, her postintervention evaluation measurements are not available. It should also be noted that because of technical issues linked to the temporalization of the interactions, participant B skipped the interaction of week 2, participant A skipped the interaction of week 3 (Figure 1), and participant D skipped the interaction of weeks 3 and 5. Furthermore, all participants had to autonomously prompt the interaction with Juno at least once.

**Table 1:** Participants’ demographic information.

Participant	Age (y)	Living area	Education level	Occupation	Marital status	Gestational week
A	29	North Italy	Master’s degree	Freelance worker	Married	18
B	34	Central Italy	PhD	Freelance worker	Married	12
C	31	North Italy	Bachelor’s degree	Employee	Cohabitant	12
D	33	North Italy	PhD	Researcher	Married	25
E	40	North Italy	PhD	Freelance worker	Cohabitant	12

### Differences and Similarities Across Cases: Questionnaire Scores

Regarding the trend of change between the 2 time points, women showed comparable levels of psychological symptoms, BA, and environmental reward at baseline, which instead seem slightly different at the postintervention time point. More specifically, participant C stands out as the only one showing a reduction in all psychological symptom variables, with changes ranging from 3 to 4 score points. However, the levels of BA and environmental reward appear seemingly unchanged. By contrast, participant A seems to exhibit a peak in the reduction of stress symptoms and an increase in BA. Interestingly, participant B demonstrated a trend of increase in anxiety symptoms, alongside a trend of reduction in depression symptoms and a notable peak in increased BA.

**Figure 3:**
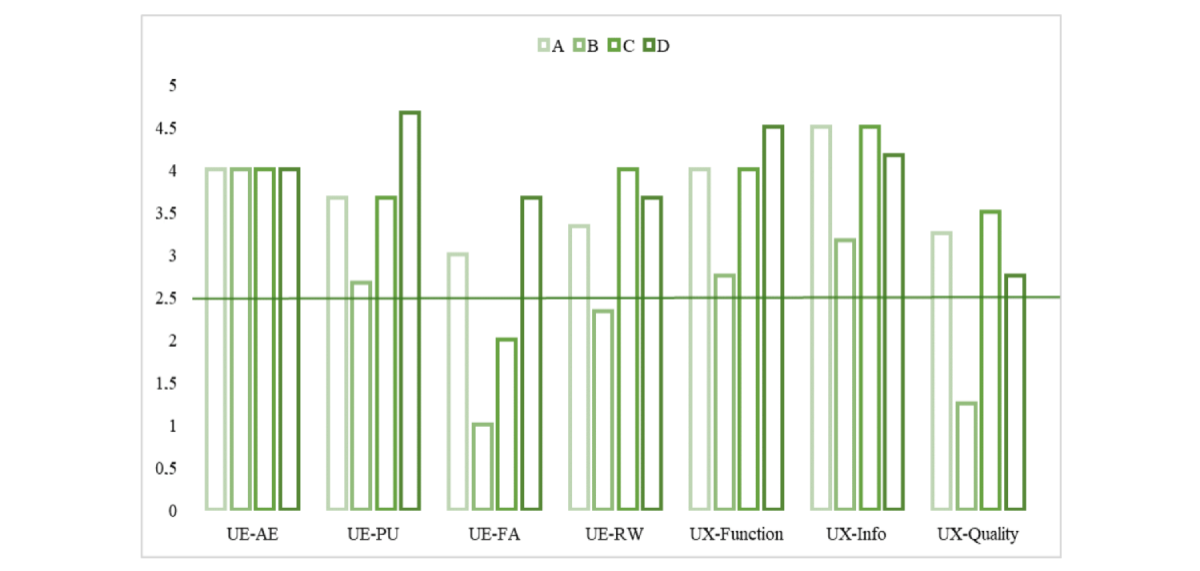
User experience (UX) and user engagement (UE) bar plot. AE: aesthetic appearance (UES-SF tool); FA: focused attention (UES-SF tool); Info: information (MARS tool); MARS: Mobile App Rating Scale; PU: perceived usability (UES-SF tool); Quality: subjective quality (MARS tool); RW: reward (UES-SF tool); UES-SF: User Engagement Scale-Short Form.

In contrast, participant D appears to demonstrate a negative peak in BA (ie, a decrease), while the other dimensions seem unchanged. Notwithstanding, it should be stressed that at either time point, none of the participants reported clinically relevant symptoms in terms of depression, anxiety, and stress symptoms. Finally, Figure 3 plots the participants’ evaluation of UX and UE. Taken together, participant B provided, in all dimensions, the lowest UX and UE scores, while participant D had the highest scores. More specifically, all showed a quite high appreciation for the esthetic of the interactions (mean 4, SD 0) and a modest to high perceived usability of the chat (mean 3.67, SD 0.82), which although seems particularly true for participant D, while less so for participant B.

Furthermore, the latter reported a particularly low sense of absorption during the interaction, which was quite low also for participant C. This sense of absorption was instead moderate for participants A and D (mean 2.42, SD 1.17). These 2, together with participant C, also reported a moderate to quite high sense of reward from the interactions (mean 3.33, SD 0.72), instead lower for participant B. A comparable pattern emerged regarding UX-information (mean 4.08, SD 0.63); in addition, participant B, for whom the information was of modest quality, participants A, C, and D instead evaluated them as high-quality information in terms of credible sources, quantity, and clearness. An almost equal score distribution emerged for the app-specific function (ie, the app operation in terms of easy learning, logical flow, and gesture interaction design; mean 3.38, SD 0.75) and subjective quality (ie, the actual availability of using the app; mean 2.69, SD 1.01), with the latter being way lower.

Multimedia appendix 1 shows participants’ specific scores reported in Figures 2 and 3.

### Differences and Similarities Across Cases: Qualitative Evaluation of the Semistructured Interviews’ Answers and Text-Mining Results

#### Overview

A summary of the key concepts that emerged from the answers provided during the semistructured interviews is reported in Tables 2-4, separately for each case. In this regard, it is worth noting the answers provided for q00 regarding the motivation for participation; only participant B reported a more personal motive linked to a desire to enrich her pregnancy experience. Differently, participants C, D, and E (participant E revealed during the semistructured interview that she is a perinatal psychologist and that she participated in the study because she was curious to experience firsthand the potentiality of digital tools in this context) were pushed by curiosity and a personal propensity to help with research. Finally, participant A reported that her curiosity was sparked by seeing one of the institutions that is part of this study.

**Table 2:** Questions and answers key points: personal experience.

Interview questions		Participant A	Participant B	Participant C	Participant D	Participant E
q00	What motivated your participation in the study? (*verbatim responses*)	“Curiosity; seeing that the Fondazione Bruno Kessler was involved in it I got curious and wanted to try and participate”	“The word pregnancy triggered my interest... I was interested in the experience itself”	“I wanted to help... and I was curious”	“The propensity to be able to be useful... when I become aware of research projects, I am pleased to be able to give my contribution.... Self-interest perhaps”	“Curiosity; because I’m a perinatal psychologist, working with mothers, and therefore having the experience of pregnancy and postpartum path and knowing the psychological aspects related to this, I was curious to see what could be done at the digital level in a sense”
q01	How have the technical issues encountered made you feel?	Interrupted the fluidity of the intervention	Confused Sorry or displeased	Indifferent, because “I knew that it was part of a research project”	Bothered Disappointed	Frustrated
q02	Can you briefly list which were the aspects that you liked the most and those that you liked the least of the intervention? Please, provide reasons for them.	Pros: Content of the exercises; Possibility to reflect Cons: Exercises were not structured enough; Perceived lack of continuity between the weeks	Pros: The initial psychoeducation; The guidance allowed by the videos and exercises Cons: The confusion related to the chatbot issues	Pros: The content; How the content was deployed Cons: The limited possibility to interact, resulting in lack of exchange and personalization	Pros: The content; How the content was deployed Cons: Some questions felt redundant	Pros: The initial psychoeducation; Clear and simple language; Directness and specificness of the contents Cons: The technical issues
q03	How would you define the content of the interactions concerning the period of pregnancy?	Pertinent (psychoeducation) Generalizable (the intent of the exercises)	Adequate Useful given the early pregnancy period	Not currently useful, but would have been useful in an earlier period of the pregnancy	Sounding Useful; allows to reason on new things and to individuate and focalize on the problem area	Pertinent Adequate
q04	Do you think that the contents you viewed and what can be learned from them can be useful to you in the future, during the postpartum, and even afterward? Why is that?	Yes. It provides insights into a new way to reason and how to question yourself	Yes. It allows you to focalize on the problem area and ask for help in general and in the postpartum period	Yes. The content on how to ask for help and what is transversally helpful	Yes. It can be transversally applied to life	Yes. It allows one to become more self-aware and so to use one’s resources to better deal with difficulties and crises
q05	How would you define or have you perceived the length of the intervention?	It was a nice accompaniment, not effortful. Given the length of pregnancy, it was preferred to be longer (“A drop in the ocean”)	It felt a bit “concentrated” because it skipped a week and the participant would have liked for it to be a bit longer or with the possibility to add a couple of weeks	Overall adequate. The interactions’ length was also adequate, not burdensome because one can answer when they can (ie, no need for an immediate response)	6 weeks would be adequate, but because it skipped a few weeks, it felt too short	Adequate, but the participant would have liked for it to be around 8 weeks

**Table 3:** Questions and answers key points: user experience and user engagement with the chatbot Juno.

Interview questions		Participant A	Participant B	Participant C	Participant D	Participant E
q06	Overall, what do you think about the interactions with the chatbot Juno?; probing questions: 1. How did you feel during the interactions; 2. What did you think of the esthetic of the material?	Easy to use and intuitive Sense of positivity, welcome kindness, and availability Makes you forget that there is not a person “on the other side” The buttons that allowed the participant to answer and continue the interaction made the interaction smoother The esthetic of the material was cute, pleasing, and unique, with its own “identity”	The technical problems made it confusing; they gave the perception of not having control over the situation and being impotent Without the technical problems, the interactions would have been positive but anyways sterile The esthetic of the material was very pleasing and able to hook the person’s interest	Not personalized enough Frustrating and information was felt as less impactful when the chatbot answer was not well in tune with the input provided The esthetic of the material was cute and pleasing	Sufficiently spontaneous and realistic Bugs in the interaction (eg, sending the same message twice) were felt as bothering	Felt followed by the chatbot Feeling that the chatbot was a virtually created entity was reassuring given the context The materials’ esthetics were cute, light, and “activating”
q07	What do you think about using Chat interactions with a chatbot to communicate content related to psychological well-being such as that Juno sent you?	The limited possibility to interact and receive feedback from the chatbot is both a strength (greater control over what is happening) and a limitation (reduced personalization) Efficient tools to do practical exercises that can foster self-reflection	Can be valid tools, but a telephone interaction with a person should be integrated in case both technical and (particularly) emotional issues might arise Because psychological content might activate something within the person, direct human monitoring is felt as a need (eg, twice during the 6 weeks)	It can be a good start that allows self-reflection, to then seek in-person psychological support	Efficient for its purpose Convenient and useful To be used only in preventive contexts in which there are not particular issues and conditions	Good idea, because it allows the participants to keep up with the increasingly technological times Allows to save time and money and through this might allow to reach people that would not otherwise get in touch with psychological contents
q08	If you could change or suggest changes, what would change your interactions with Juno? That is, beyond the content of the messages, what would remove and add to the way Juno interacts?	Fix the chatbot bugs Make the interactions more personalized	Better inform the user of the potential technical problems and provide information on how to autonomously navigate them to reduce the sensation of confusion	Receive information based on the level of interest in knowing about a topic More in-depth suggestions in favor of well-being	Nothing, beyond fixing the chatbot’s bugs	d/n^a^
q09	What do you think about the use of Telegram as an app through which to communicate with Juno, or anyway, with a chatbot?	Favors the perception of personalization because seeing Juno within the other Telegram chats makes it feel like a person (“I felt as if she was always in my thoughts”)	It is good not having to download another smartphone app, it makes interacting with the chatbot more comfortable	It is good not having to download another smartphone app, the interaction was perceived as more immediate	It is good not having to download another smartphone app, it was convenient to use Telegram	It is an appropriate means because it is easy to use and does not require downloading additional applications, making it more likely to be utilized by those less comfortable with using new technological tools

^a^d/n: did not know what to answer.

**Table 4:** Questions and answers key points: opinions on future technological advancements.

Interview questions		Participant A	Participant B	Participant C	Participant D	Participant E
q10	Based on your pregnancy experience, if you could imagine an ideal app that would provide you with psychological support, how would it be? probing questions: 1. How would you like the information to be structured and provided? 2. Concerning the ease of use and the clarity of the commands, how important do you think they are? What would make it clearer or easier to use? 3. What kind of content would you like to see?	An app providing 360° support during pregnancy in the form of: Informative, scientifically supported psychological content structured in fixed modules that can be consulted at a time of choice Provide applicative suggestions Support self-screening of psychological and physical symptoms to help them understand when it is needed to ask for professional help Personal diary to write medical and psychological information An always-available chat to ask questions and have specific feedback to receive support on one’s personal experience Clear and simple app structure and organization with visual cues	Informative content (medical and psychological) in the form of fixed modules Optional chat that provides specific interventions to support well-being Clear and simple app structure and organization FAQ^a^ section on how to manage technical problems	A chat that provides weekly information on how to read bodily sensations throughout the pregnancy to understand what is “normal” and what is not A more personalized chat in which questions are asked about eventual anxiety A simple chat with few requirements	Informative content (medical and psychological) in the form of fixed modules: what is normal, what is not, and what to expect Content divided by pregnancy period A flexible and more personalized chat in which questions are asked about specific feedback to receive support on one’s personal experience Clear and simple app structure and organization	Informative content (medical and psychological) in the form of fixed modules: what is normal, what is not, and what to expect Information on eventual miscarriages and how to deal with and elaborate on it Includes information on services and health professionals in the surrounding area A flexible and more personalized chat in which ask questions Clear and simple app structure and organization
q11	What technological aspects would it add to a tool like Juno? probing questions: 1. What do you think about voice commands and voice responses from Juno? 2. Would you like to customize the look of the chatbot? If so, in what terms?	Increased personalization in the chatbot’s feedback, with the chatbot connecting information across the interactions More visual content Do not feel the need for voice commands Personalized avatars to increase the immersion	Increased personalization in the chatbot’s feedback to give the perception that what was said is being understood and for it to make connections on things said across the interactions Do not feel the need for voice commands (reduces the privacy) or to personalize the chatbot appearance	Increased personalization in the chatbot’s feedback and in the capacity to understand inputs Do not feel the need for voice commands (reduces the privacy) or to personalize the chatbot appearance	Increased personalization in the chatbot’s feedback. to create a more realistic and immersive experience Do not feel the need for voice commands or to personalize the chatbot appearance	Voice commands to easily interact with the chatbot while doing other activities Increased personalization to render interactions more realistic Possibility to personalize the esthetic features and create an avatar to make the experience more enjoyable
q12	Is there something that worries you about using technologies like chatbots and smartphone apps as tools to provide psychological support?	The idea that these technological tools can substitute in-person professional support when they should be used only for an initial self-refection to then open the way for in-person and structured support	That proper support cannot be provided when the psychological content provided opens up some internal matters that should warrant professional attention Lack of monitoring of the person’s well-being Lack of monitoring and control over the chatbot’s behavior	Eventual bugs and wrong information provided by the chatbot Reduced human control of the chatbot behavior and the information provided	Risk related to an increase and excessive reliance on technological means. These tools can be of support, but should not substitute interaction with professionals	Risk related to an excessive reliance on technological means, untimely substituting in-person professional support Eventual wrong information provided by the chatbot
q13	What do you think might be the pros and cons of using technology over human support in providing psychological support during pregnancy? probing question: 1. Do you think there are personal or social situations in which one can be more suitable than the other?	Pros: The asynchronicity allowed by chat interactions; Allows for greater freedom; It can provide more direct indications on where to autonomously look for information Cons: It cannot understand and give feedback based on the human “gut feeling” but remains at a more rational level of understanding; It cannot be effective for specific issues but remains more general	Pros: Capacity to reach a broader part of the population; Easy to access Cons: Might lead to the idea that it can substitute in-person professional support	Pros: Easy to access and readily available Cons: Being and feeling lonely; Difficulty in maintaining attention and focus because of a lack of in-person guidance and support; Modulating the interaction based on the psychological and emotive state of the person and eventual changes of these during the interaction	Pros: A first hint to favor self-reflection; Readily available to anyone; Can be a first attempt at seeking support among those more ambivalent about it; It can work as a momentary buffer in contexts in which one cannot physically move; If smartphone based, it is readily available Cons: It cannot (nor should) match the support that can be provided through in-person contact; Cannot provide the benefit and warmth of human contact	Pros: Easy to access, readily and constantly available; Might allow to contain moments of heightened anxiety; Can provide support to those with reduced economic possibility; Might serve to “plug” gaps in the support provided by the health system; Might create the self-awareness needed to seek support when it’s needed Cons: Excessive reliance on the tool, untimely avoiding seeking professional support
q14	In your opinion, what could be done or created to manage the challenges and risks that you have mentioned to support the reliance on and the use of these technological tools?	Inform the person on what to expect and not to expect from the digital tool so as not to feel disappointed and demotivated when the feedback does not match expectations Underline and remind that the tool cannot substitute in-person professional interactions	Information on what and what not to expect from the digital tool (ie, the limits of the technological tools) Remind that the tool cannot substitute support from professionals FAQ on how to manage technical problems to avoid the feeling of loss of control over the tool	Incorporate a form of screening or monitoring that can alert both the individual and professionals to the need for further evaluations and increased support	Underline and remind that the tool cannot substitute in-person professional interactions Incorporate a form of screening or monitoring that can alert both the individual and professionals to the need for increased support	Underline and remind that the tool cannot substitute in-person professional interactions Included scientifically sound information and report them as such
q15	What do you think about the idea of integrating this type of tool within the health system and/or routine care with your gynecologist to promote the psycho-physical well-being of pregnant women?	Useful and needed Can support the trustworthiness of the tool and the information provided, thus increasing the likelihood of it being used	Useful and needed More monitoring that is easier to conduct Can give a sense of continuity between visits and of being more broadly taken care of Can bridge and ease interactions with medical professionals Can favor the trustworthiness of the tool and the information	Useful as it might allow for increased and more constant monitoring of the psycho-physical well-being of the woman	Useful and needed; it can give a sense of continuity between visits and of being more broadly taken care of	Useful and needed to compensate for gaps in the health care system Can favor the trustworthiness of the tool and the information provided
q16	Is there anything else you would like to add?	—^b^	—	—	Include a joint and/or separate intervention for the partner and people in the women’s support network	Include information more specific to the postpartum period in the interactions with Juno Having a support intervention specific for the postpartum and related challenges that reminds the woman to take care of herself

^a^FAQ: frequently asked question.

^b^Not applicable.

**Figure 4:**
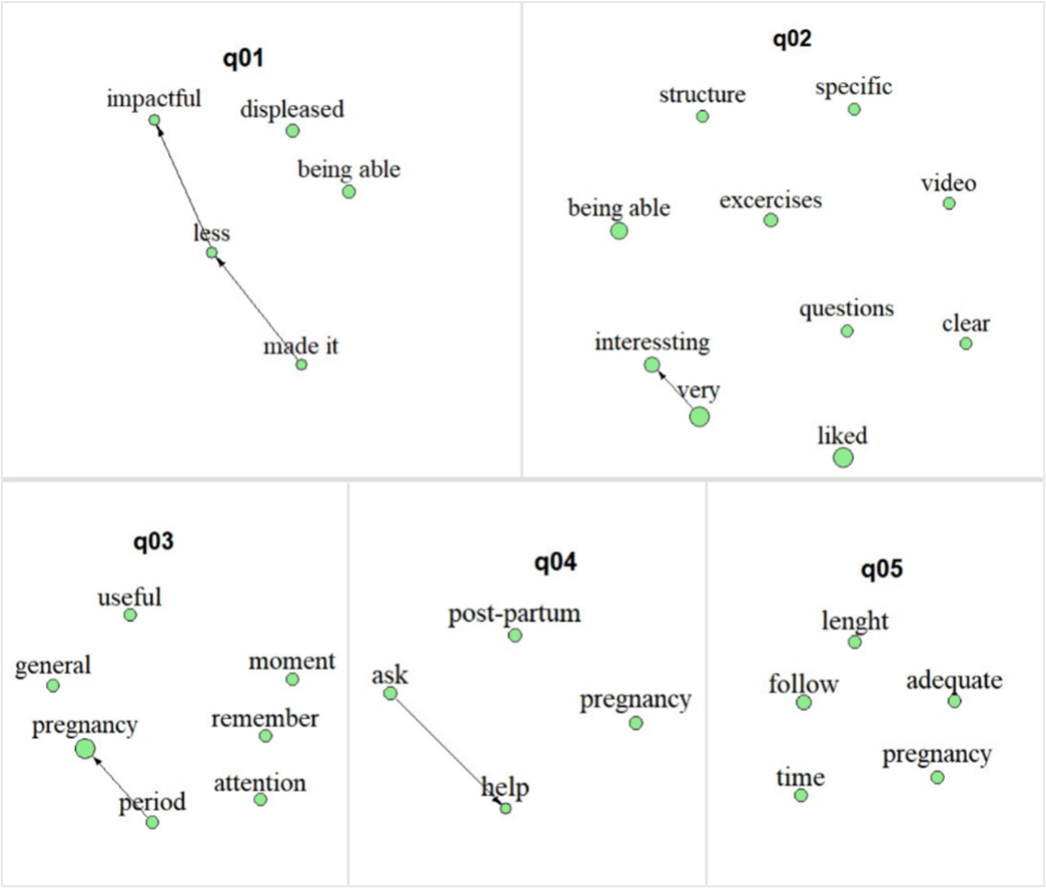
Text-mining results: answers regarding personal experience.

**Figure 5:**
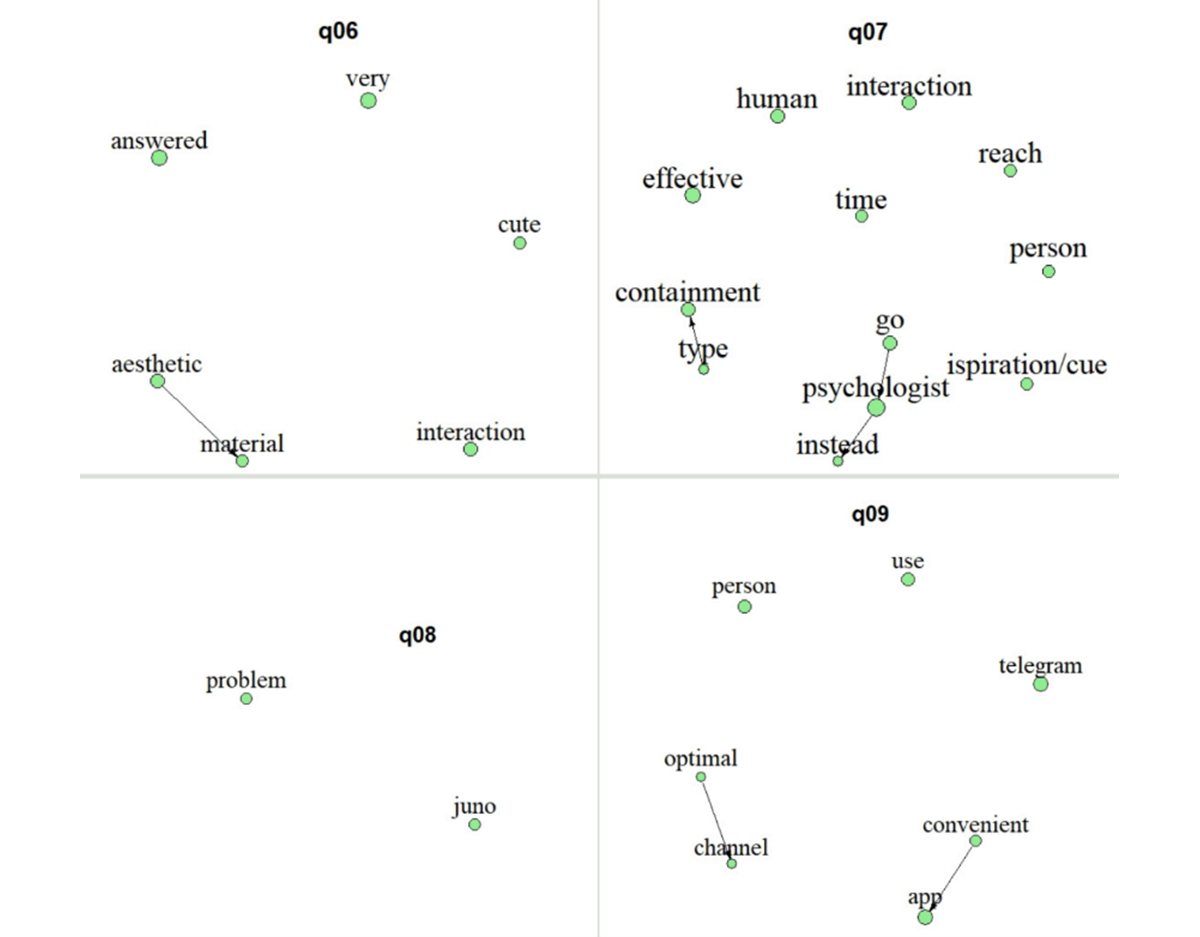
Text-mining results: answers regarding user experience (UX) and user engagement (UE) with the chatbot Juno.

**Figure 6:**
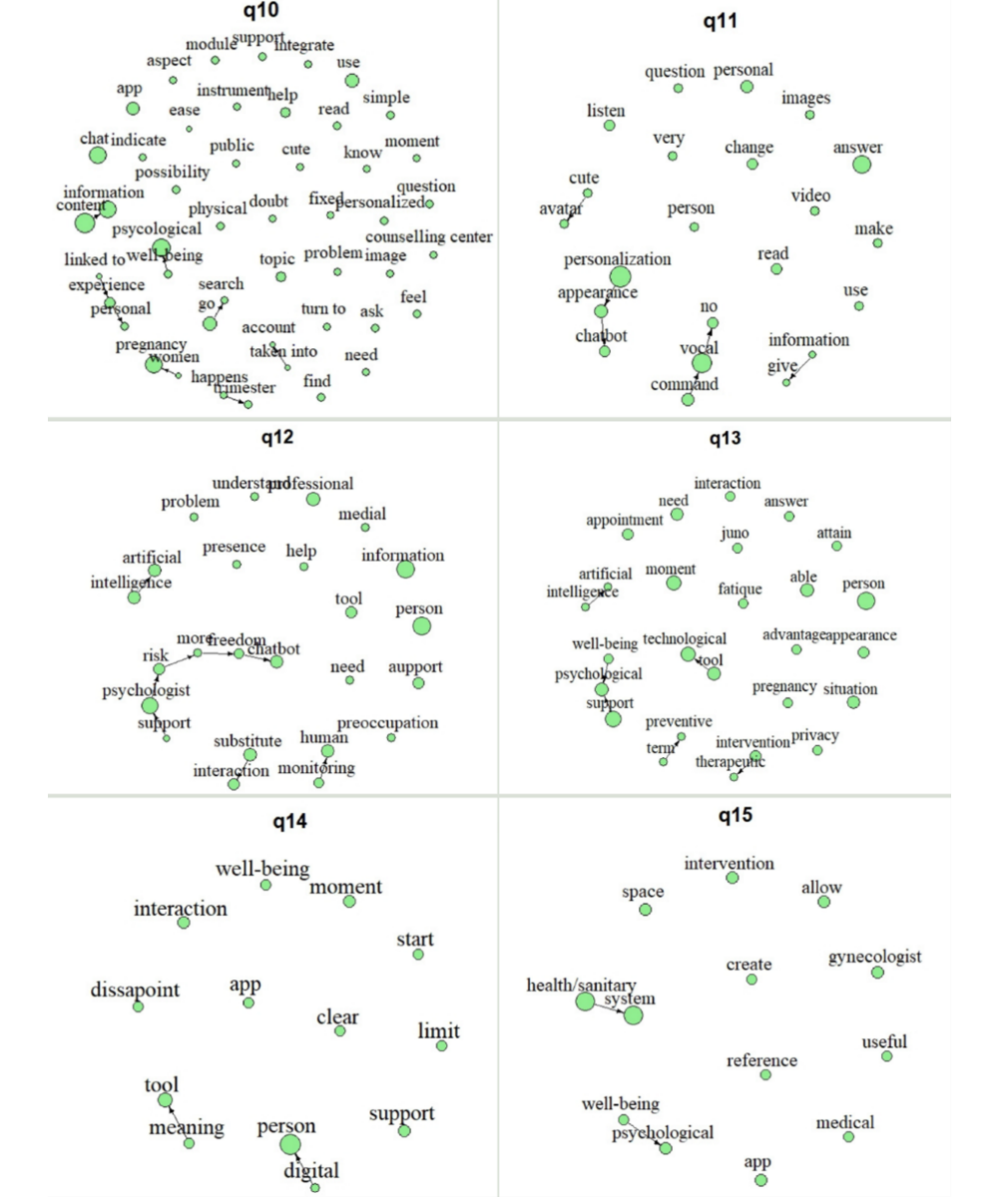
Text-mining results: answers regarding opinions on future technological advancements.

Focusing on the text-mining analysis performed, the interview length ranged between 27.41 and 60.02 (mean 42.3, SD 12.63) minutes. After deleting the stop words, transcripts included a mean of 1732.8 (SD 795.78) words per participant. Overall, the aggregated results (text mining) are shown in Figures 4-6. The nodes (ie, word stems) dimension illustrates the proportion of concept occurrences across transcripts for a specific question, all appearing at least 3 times. Word stems connected by arrows represent diagrams that have occurred at least twice. The word stems are translated after analysis for inclusion in the plots; therefore, the direction of the arrows reflects the Italian syntax.

#### Personal Experience With the Chatbot Juno

As for the text-mining aggregated results, the transcript of the interviews regarding participants’ personal experience with Juno included, after deleting the stop words, a mean of 67.56 (SD 28.39) words per question. Results are summarized for each question in Figure 4. Instead, Table 2 summarizes, separately, the participants’ answers to each question. In this respect, all participants reported feeling negative regarding the technical problems encountered, except participant C, who felt indifferent to them. Noteworthy is that although all skipped at least 1 interaction, participant C is the sole one that had not skipped any interaction, while further reporting that she “knew that this is a research project,” thereby highlighting that she had foreseen some issues to occur. In line with this, the text-mining results highlighted the feelings of *displeasure*, untimely making the experience *less impactful* (*made it→less→impactful;* q01).

Nonetheless, the experience (in terms of the content of the interactions) was *liked* and felt *very interesting*, in particular, the content of the *exercises/questions* (q02). However, participant A specified that she would have preferred if the latter were proposed in a more structured manner while also allowing for the possibility to continue practicing them in between the interactions to favor a sense of continuity. Furthermore, participant D felt that some of the questions (part of the exercises) were redundant. Participants B, C, and D instead stressed their appreciation for how the broader content was deployed in terms of videos and images, while participant E specifically appreciated how the messages were phrased. The overall content of the interactions, particularly the initial psychoeducation, was felt as pertinent and adequate. Coherently, the text-mining results highlighted that the interactions’ content was felt *useful*, allowing participants to take a *moment* to pay *attention* to themselves (q03). In this regard, they all reported that the content was pertinent to the pregnancy period (*period→pregnancy*) but could also be useful during the *postpartum* and the future in *general*, supporting them in asking for help (*ask→help*; q03 and q04) and in general favoring a self-awareness that can transversally be applied to life in favor of well-being. However, focusing on the subjective answers, while participant B felt that the content was suited for the beginning of the second trimester, participant C felt that such a period was already too late and that the support provided by the chatbot was better suited for the emotional tumult of the first trimester.

At last, all women felt the 6-week length of the intervention was *adequate*, although, given the length of the *pregnancy* period, they could have *followed* it even for a longer *time* (q05). This latter aspect was stressed by all those who had skipped at least 1 week of interaction and not by participant C, who had followed all 6.

#### UX With the Chatbot Juno in Telegram

The transcript of the interviews regarding participants’ UX with the chatbot Juno in Telegram included, after deleting the stop words, a mean of 69.65 (SD 29.95) words per question. Results are summarized for each question in Figure 5, while Table 3 reports participants’ answers. With regard to women UX in interacting with Juno, as previously outlined, experiences were quite different, albeit the technical *problems* with the chatbot *Juno* have emerged as a matter to particularly account for (q08). In this regard, participant B pointed out the importance of providing clearer guidance, ideally beforehand, on how to autonomously deal with technical issues to help avoid feelings of confusion. Notwithstanding, all women showed appreciation for the esthetic of the material (*esthetic→material*) describing it as *cute*. Furthermore, it mostly brought the focus of the UX to the way Juno *answered* their inputs, highlighting the relevance of this aspect, thereby wishing for an increased personalization of the answers (q06). However, despite this, participant A perceived that because of the way messages, in general, were phrased and of the overall interaction flow, these made her at times “forget that there was not a person on the other side.” This is instead different from participant B’s perception, who considered the messages to be a bit sterile. In between these 2 polarities is instead the perception of participants D and E, the former describing them as “sufficiently spontaneous and realistic” and the latter further stressing that, although she felt properly guided by Juno, perceiving clearly that Juno was virtually created made her feel reassured. Coherently, when asked about their opinion on using a chatbot as a means to deploy psychological content (q07), participant A reported that the interactions’ limits (in terms of chatbot freedom) were both a limit and a strength. Nonetheless, overall, women felt that it could be an *effective* medium that can provide a kind of momentary containment (*type→containment*) and that it might work as a *cue* to subsequently *reach* for in-*person* support. Indeed, they felt that beyond its application in preventive contexts, a psychologist is needed (*go→psychologist→instead*; q07), and even in the context of this study, participant B felt the need for human contact at least by telephone call.

Finally, focusing on the app itself, women all agreed on the convenience (*convenient→app*) of Telegram as an interface, allowing them to avoid downloading another app and describing it as an *optimal channel* that they already knew and that is easy to *use* (q09).

#### Opinions on Future Technological Advancement

The transcript of the interviews’ answers regarding the participants’ desired technological advancement included, after deleting the stop words, a mean of 159.49 (SD 53.01) words per question. Results are summarized for each question in Figure 6; Table 4 reports the participants’ answers. Overall, when asked about opinions on future technical advancements, women’s answers were quite cohesive. In line with this, when asked about how they would image an ideal app in the context of perinatal care (q10), the greater focus was on the information content (*content→information*) related to what happens during *pregnancy* and in the different *trimesters* (*happens→trimester*) as well psychologically (*well-being→psychological*). It was also focused on the possibility of searching for this information and reading about it (*go→search*) freely.

Furthermore, they reported interest in having a *chat* with a chatbot within the app mainly to ask personal *questions* related to their personal experience (*linked to→experience→personal*). In this regard, focusing specifically on the potential technological advancements that could be foreseen for chatbots like Juno (q11), women showed a lack of interest in including vocal commands in terms of sending and receiving audios (*command→vocal→no*) and did not show a particular interest in personalizing the chatbot appearance (*personalization→appearance→chatbot*), albeit recognizing that others might. The sole exception was participant E; she reported voice commands as the first thing she would have liked to add, perceiving it as a way to optimize time. Regarding *personalization*, this aspect was again prominent among all women, stressing their desire to receive more personalized (*personal*) *answers*. However, albeit desired, such increased personalization and freedom of the chatbot also emerged as women’s main concern regarding the application of these tools in the mental health context (q12). As such, women reported the need to maintain *human→monitoring*. Indeed, worries were expressed regarding the kind of *information* the chatbot might give if unsupervised.

Furthermore, they expressed worries related to increased freedom and resemblance to human interactions, with the idea that this might lead to an overreliance on these tools. Indeed, they stressed the risk of these substituting interactions (*substitute→interaction*) with professionals and psychologists (*support→psychologist→risk→more→freedom→chatbot*), which was not desired. In line with this, participants believed that although a main advantage of these *technological→tools* is that they can be valuable in supporting *psychological→well-being* in preventive contexts (*preventive→terms*) or to satisfy specific *needs* without waiting to make an *appointment*, they cannot equate a *therapeutic→intervention* delivered in-person, particularly during *pregnancy* (q13). To deal with the concerns and risks reported, women agreed on the importance of underlining and reminding of what to expect from these tools (*meaning→tools*) and clearly stating their *limits*, thereby distinguishing the kind of support that can be received by a physical person versus a digital tool (*digital→person*). This would then also work as a disclaimer, thus preventing them from feeling *disappointment* when perceiving the limits of these tools (q14). In line with the above, women expressed a strong desire for an *app* that could be integrated within the *health* (care)*→system,* perceiving it as something that could create a shared *space* that facilitates interactions with *gynecologists*, thereby allowing the latter to account for women’s *psychological well-being* together with the *medical* aspects.

## Discussion

### Principal Findings

This study aimed to use a multiple–case study design to evaluate and compare pregnant women’s experience and perception of Juno, a chatbot prototype to deploy a BA preventive support intervention; their opinions regarding desired improvements and technological advancements were also investigated. The insights gained from this study are valuable and in line with previous studies emphasizing the importance and essential nature of evaluating prototypes during the design stages of a digital tool and chatbot in particular [41,63]. Within this context, the adoption of a multicase study design [42] allowed us to gather valuable in-depth information on the similarities and differences in pregnant women’s perceptions, opinions, and desires while also evaluating the technical issues encountered and their impact on women’s experience [63].

Focusing on the implementation and operationalization of Juno in Telegram allowed women to benefit from the lack of installation requirements, experiencing an interface within a familiar environment; this is an advancement from the platform used in the previous study [36]. Instead, feedback regarding the materials’ esthetic and intervention content at large was again appreciated, and the content, in particular, was described as sound and useful. Women expressed specific appreciation for the exercises proposed by Juno as part of the BA intervention, assessing that they favored self-reflection. Differently from the previous study [36], most women would have liked for the intervention to be longer. This might be linked to the weeks of interaction skipped since only participant C who had completed all 6 interactions would have not lengthened the intervention. Another explanation could, instead, be linked to the change of platform and even more the new structuring of the exercises. Compared with the previous structuring [36], they have been changed so to be as short, simple, and effortless as possible, and as such, they were turned into on-the-moment reasoning exercises guided by Juno and no longer as in-between–session homework. This altogether seems to have been appreciated by women, except for 1 participant A, who instead stressed that she would have preferred to have the possibility to continue training them autonomously through practical exercises also in between the interactions. The desire for continuity became evident throughout her interview, suggesting that the digital tool was perceived as a companion to turn to when extra support was needed by providing a personal space to freely take care of herself. Participant E also highlighted this, considering it as something with added value, especially during the postpartum period, helping her take care of herself to then potentially better care for the newborn.

Mindful of the above, it is pivotal to remember that women in this study can be deemed “healthy,” as none of them present medical conditions, and all psychological symptom variables were below the clinical thresholds. Notwithstanding, it is worth noting that participant C, who besides having cleared the 6 interactions, also reported the highest symptomatic scores at baseline, showed the greatest and most consistent trend of reduction in symptom scores. However, despite some pretest-posttest changes in symptom variables scores, none of the participants ever reached clinical relevance, so they should be regarded as normal fluctuations in the state of well-being and ill-being occurring during pregnancy [64]. As such, the positive feedback on the meaning of the content and the exercises proposed is important since within a preventive context, the goal is not symptom reduction or resolution but to emphasize the awareness of psychosocial functioning and the intricate relationship between emotions and behaviors. This would ultimately allow the development of transversally applicable personal resources that can be applied across life situations, thus fostering adaptation capacities at large. Women here appeared to have perceived these benefits, acknowledging that the intervention content was relevant to the pregnancy period and could potentially be helpful during the postpartum and in the future. However, it is worth highlighting a difference in its perceived usefulness as a function of the pregnancy period during which they thought that the intervention should be deployed. Participant B considered it suitable for the early pregnancy period, around the beginning of the second trimester, while participant C suggested it was more beneficial for the emotional tumult of the first trimester. It is noteworthy that both women followed the intervention during the same gestational week. Such individual differences in the perception of need are though important in terms of motivation in following the preventive intervention and in the foreseen impact of the information received.

Notwithstanding these individual differences, consistent patterns across women’s feedback were identified, particularly when asking them how their ideal digital tool developed to provide psychological support during pregnancy should be. The first thing they stressed regarded the content; within a preventive context, all women felt the need for more holistic information in which the medical and the psychological aspects are integrated, helping them understand how the 2 influence each other to then receive guidance in understanding what is “normal” and what is not. They expressed a preference for this information to be readily available, giving them the freedom to access it as they preferred. This could indeed support their empowerment [26] and aligns with the evidence highlighting that engaging in help-seeking–related behaviors increases the likelihood of perinatal women seeking further assistance in the future if needed [28]. As for receiving support for their own subjective experience, women pointed to chatbots, seeing them as having the potential to provide a 24/7 means to answer their pregnancy-related questions. This was viewed in the context of containing worries without the need to wait or continually seek assistance for potentially smaller concerns. However, all women consistently emphasized that chatbots should not be intended to substitute in-person support or human relationships more broadly. They highlighted the importance of the perception of contact and vicinity for pregnant women. Furthermore, although recognizing the potential benefits in preventive contexts, in situations of greater need and increased psychological symptoms, women stressed that chatbots should always be accompanied by human monitoring.

Keeping this in mind, it is important to reason about the technical problems encountered with the chatbot Juno. Except for the sole woman who did not skip any interactions (participant C), all other women reported their dissatisfaction with the technical problems encountered. Their reactions varied from feeling that the intervention content became less impactful to experiencing frustration, disappointment, confusion, and a perception of loss of control. These aspects are particularly significant in a mental health context, even if preventive, since such perceptions might reduce the willingness to follow the intervention by hindering their sense of agency. Notably, participant B, who participated with the desire to enrich her pregnancy experience, reported higher and more personal expectations toward the intervention. Consistently, her scores on the UX and UE questionnaires and the results from the semistructured interview suggest that she felt the most negative about the technical problems encountered. In contraposition, participant C, who assessed that she knew that interactions with Juno were “part of a research project,” acknowledged the technical problems but remained indifferent. However, it is crucial to consider the reported feelings of confusion and loss of control. While desiring a more personalized chatbot to receive answers that are more in tune with their individual needs, participants themselves emphasized the importance of clear explanations and reminders about the chatbot’s capabilities and limitations as it becomes more sophisticated and autonomous. This is essential to prevent overreliance on the chatbot and to avoid potential disappointment and iatrogenic effects that could decrease the likelihood of seeking help in the future. When discussing the use of chatbots like Juno as a means to deliver psychological content, participants acknowledged its effectiveness in providing momentary containment and serving as an initial step before seeking further support. However, they also underscored the need for human psychologists or professionals in preventive contexts. Participant B, in particular, expressed a desire for some “human” contact, even by telephone while interacting with Juno. This resonates with a compelling argument made by Sedlakova and Trachsel [40]; they conducted an epistemic analysis of chatbots’ adoption within mental health or therapeutic contexts, prompting the need to carefully reason about how chatbots can be perceived. As such, in line with women’s desire for increased chatbot personalization but worries linked to its potential increased freedom, the authors [40] suggested balancing the number of humanlike characteristics and features of chatbots and that their application should be confined to specific functions.

Focusing on the broader real-life application of apps and chatbots in contexts such as the health care system, beyond being highly desired, they were seen as tools that could bridge between women and clinical professionals. Moreover, in line with the above, women reported that having such tools would make them feel like their psychological well-being was accounted for together with their physical well-being since the former is felt neglected. Another aspect that has emerged is that they could help favor self-monitoring and monitoring from the clinician; existing literature does indicate that tools of this nature are acceptable to perinatal women as a means of monitoring mood symptoms [65]. In this regard, the Interactive Centre of Perinatal Excellence developed by the Australian Centre of Perinatal Excellence [66,67] is noteworthy. It is an interactive digital screening app integrated into the health care system and designed to facilitate screening for perinatal depression and anxiety symptoms. It provides women with feedback on the screening results while generating related reports for the clinician. It can support the prevention of perinatal mental health disorders by empowering women, streamlining the screening process, and saving time and resources for both women and clinicians. In such a context, the inclusion of a tool like Juno within an app to “educate” and guide women through their pregnancy and postpartum while allowing for symptom monitoring might hold great potential; *what if, within such an app, the preventive BA intervention deployed through Juno was proposed to women showing mild and/or moderate depression symptoms?*

The literature highlights consistent prevalence metrics of depression symptoms throughout the whole pregnancy period (worldwide, 20.7%; Europe, 17.9% [68]; Italy, 6%-22% [69-72]), pointing at it as among the main predictors of postpartum depression [16,73], with repercussions on the quality of life of women [74] as well as on the child’s development and well-being [75-77]. These metrics highlight the necessity of collaborative efforts in designing and implementing tailored programs, particularly in primary prevention (to prevent symptoms before they start) and secondary prevention (targeting individuals at risk or with subclinical symptoms) [78]. This is especially crucial, given the unique characteristics of peripartum depression, referring to its direct association with the challenges and bodily changes inherent to the perinatal period [79] and stressing the need for tailored intervention programs. In this regard, taken together, our results suggest that Juno holds potential for apps in a preventive context, which is of value considering the paucity of preventive perinatal tools [34]. However, it has also emerged that within this context, a tool like Juno is not deemed as sufficient. In this regard, the comments made by participant A are emblematic; she felt that what Juno could give within the broader perinatal period was like “a drop in the ocean.” This is further exemplified by the dropout of all women with medical conditions, which suggests that in its current form, the intervention deployed through Juno would have limited application. As such, data indicate that its real-life adoption might be scarce if not inserted within a broader context that can better signify its value while allowing us to account for women’s differences in need, which influences the type and amount of use they would make of it. Furthermore, beyond ensuring a better functioning of the tool itself, a thorough action plan linked to problem resolution should be defined and provided to women (both in research and real-life contexts). However, the evaluation of these issues resonated with literature emphasizing the advantages of incorporating dedicated prototyping and implementation phases during the co-design of digital tools [41].

### Study Limitations and Future Directions

Although the results’ generalizability cannot a priori be expected in this study design, women’s high educational level and residency in northern Italy still represent a limitation. Perinatal depression symptoms tend to be higher among women with lower educational levels [80], suggesting a potential bias in the sample. In addition, the mentioned sample’s characteristics may reduce the variability of analyzed cases, impacting the generalizability of the findings. A further limitation regards data collection, as it relied on self-reports and semistructured interviews, which are indeed vulnerable to social desirability biases. Moreover, in this study, women’s experience with depression symptoms and their use of e-mental health tools were not measured, thus representing a limit of the study. Nonetheless, assessing these matters could provide valuable insights into their perceptions and potential use of chatbots during pregnancy. These dimensions warrant consideration in future studies to better understand the factors influencing women’s engagement with digital interventions during this critical period.

Being at the beginning of the product life cycle [45,46], referring specifically to the technical problems encountered, while they represent a limitation in the study, their management by the research team was invaluable in providing insights into the software used and the potential of Rasa. This understanding contributes to a more flexible problem-solving approach for addressing current and potential future issues. Proactively addressing such problems helps users maintain a sense of control and proficiency with the tool. In addition, these issues offer important information on how problem resolution, or lack thereof, impacts the overall UX. In this regard, it is noteworthy that the users did not express dissatisfaction with the simplicity of the solution, which primarily operated on rule-based mechanisms. This emphasizes the significance of incorporating user-centered design principles in developing natural language processing solutions that effectively meet end users’ needs and expectations.

### Conclusions

In line with this, good practices can be outlined to construct appropriate validity and mitigate any negative effects on the user, thus ensuring ethical standards [81-86]: (1) ground intervention content in evidence-based data pertinent to the perinatal literature; (2) seek input from both end users and clinical professionals to evaluate needs and gather feedback on intervention content and e-mental health tool usability; (3) conduct feasibility and pilot-testing to ensure acceptability, feasibility, efficacy, and effectiveness, along with evaluating e-mental health tool use (both frequency and duration); (4) use adequate measures and evaluate appropriate outcomes to assess intervention success; (5) ensure that end users are provided with a clear informed consent regarding intervention purpose, content, and e-mental health tool capacities, risks, and limits; (6) incorporate safety measures, including clear procedures for managing situations of need and heightened distress, such as providing crisis support services and establishing connections with reference clinicians or public health services, while also monitoring end user mental health; (7) continuously monitor intervention progress to refine effectiveness and minimize potential negative effects; and (8) ensure clinical professionals are properly guided and informed with up-to-date evidence on available e-mental health interventions, their effectiveness, suitability, and safety. Aligning with this, to ensure a consistently high-quality technical solution for end users, substantial investments in assistance and infrastructure are imperative. The insights from this study underscore the importance of prioritizing UX and technical reliability to enhance the effectiveness and adoption of preventive perinatal tools like Juno in real-world contexts. Although Juno already aligns with ethical standards 1 to 5, the results of this study indicate that the tool’s capacities, risks, and limitations need to be greatly reported (point 5). In addition, safety measures were limited to self-reported depression, anxiety, and stress levels, with no specific process for monitoring intervention progress (point 6). Therefore, future developments of Juno should incorporate comprehensive safety measures and test their feasibility and acceptability. This includes integrating technological requirements to establish a more specific procedure for monitoring intervention progress (point 7).

## Appendix

Multimedia Appendix 1 Definitions of digital intervention and technological advancement provided to participants during the semistructured interviews and the participants’ scores.

## Abbreviations

BA: behavioral activation
ORBIT: Obesity-Related Behavioral Intervention Trials
PHQ-9: Patient Health Questionnaire-9
UE: user engagement
UX: user experience
WHO: World Health Organization
