# Supplementary material for: A Chatbot (Juno) Prototype to Deploy a Behavioral Activation Intervention to Pregnant Women: Qualitative Evaluation Using a Multiple Case Study
Source: JMIR Form Res. 2024 Aug 14;8:e58653. doi: 10.2196/58653 (PMC11358662; doi:10.2196/58653)
Supplement: Multimedia Appendix 1 [file formative_v8i1e58653_app1.doc]

**Definition of “digital intervention” and “technological advancement” provided to participants during the semi-structured interview.**

*“By digital intervention we mean the use of technologies for prevention or treatment, in the context of mental health, concerning the use of technological tools as a means of communicating the contents of an intervention whose efficacy has been scientifically proven. In this context, a Chatbot like Juno is, therefore, a means through which to communicate the information of the intervention.*

*Speaking, instead, of the technological advancement of these technological tools, we mean the integration of Artificial Intelligence within these technological tools, which allows to make them more sophisticated. With Artificial Intelligence, specifically, we mean computer systems that allow a machine/a technological tool to perform tasks (both physical and cognitive) in a way similar to humans, including the detection, perception, and capacity to learn patterns of human language and behavior. In other words, they are computer systems that try to learn how human language and behavior work to then imitate it. Here we focus on language, where a Chatbot like Juno, who is an artificially generated virtual character, when supported by Artificial Intelligence technologies can be able to imitate human conversations, making them more realistic and natural in the way they speak.”*

Table S1. Participants' symptoms and activity levels at baseline (T1) and post-intervention (T6) and the difference (Δ) between T1 and T6.

|  |  |  |  |  |  |  |  |  |  |  |  |  |  |
| --- | --- | --- | --- | --- | --- | --- | --- | --- | --- | --- | --- | --- | --- |
|  | A | | | B | | | C | | | D | | | E |
|  | T1 | T6 | Δ | T1 | T6 | Δ | T1 | T6 | Δ | T1 | T6 | Δ | T1 |
| **PHQ-9** | 1 | 2 | 1 | 7 | 4 | **-3** | 5 | 2 | **-3** | 2 | 3 | 1 | 5 |
| **EPDS** | 7 | 11 | 4 | 10 | 10 | 0 | 10 | 7 | **-3** | 8 | 9 | 1 | 9 |
| **GAD** | 3 | 5 | 2 | 2 | 5 | **3** | 6 | 2 | **-4** | 6 | 4 | -2 | 3 |
| **PSS** | 8 | 2 | **-6** | 10 | 12 | 2 | 11 | 8 | **-3** | 8 | 10 | 2 | 13 |
| **BADS-SF** | 23 | 31 | **8** | 18 | 29 | **11** | 24 | 23 | -1 | 30 | 23 | **-7** | 25 |
| **EROS** | 26 | 27 | 1 | 20 | 22 | 2 | 25 | 26 | 1 | 26 | 25 | -1 | 24 |
|  |  |  |  |  |  |  |  |  |  |  |  |  |  |

***Note****. BADS-SF = Behavioral Activation for Depression Scale – Short Form; GAD-7 = Generalized Anxiety Disorder – 7; EPDS = Edinburgh Postnatal Depression scale; EROS = Environmental Reward Observation scale; PHQ-9 = Patient Health Questionnaire-9; PSS = Perceived Stress Scale;* Δ = difference between T1 and T6

*Table S2. UX and UE*

|  | A | B | C | D |
| --- | --- | --- | --- | --- |
| **UE-AE** | 4 | 4 | 4 | 4 |
| **UE-PU** | 3,7 | 2,7 | 3,7 | 4,7 |
| **UE-FA** | 3 | 1 | 2 | 3,7 |
| **UE-RW** | 3,3 | 2,3 | 4 | 3,7 |
| **UX-Function** | 4 | 2,8 | 4 | 4,5 |
| **UX-Info** | 4,5 | 3,2 | 4,5 | 4,2 |
| **UX-Quality** | 3,3 | 1,3 | 3,5 | 2,8 |

***Note****.*AE = Aesthetic appearance (UES-SF tool); FA = Focused attention (UES-SF tool); Info = Information (MARS tool); PU = Perceived usability (UES-SF tool); Quality = Subjective quality (MARS tool); RW = Reward (UES-SF tool); UE = User Engagement; UX = User Experience.
